# Pre-pregnancy BMI was associated with gestational depressive phenotypes in a population of 12,099 women in Chongqing, China

**DOI:** 10.3389/fendo.2022.1058160

**Published:** 2023-01-10

**Authors:** Yi Chen, Huayan Gu, Niya Zhou, Wenzheng Zhou, Jia Cao, Qing Chen, Haiyan Zhang

**Affiliations:** ^1^ Key Lab of Medical Protection for Electromagnetic Radiation, Ministry of Education of China, Institute of Toxicology, College of Preventive Medicine, Third Military Medical University (Army Medical University), Chongqing, China; ^2^ School of Public Health, Guizhou Medical University, Guiyang, China; ^3^ Department of Obstetrics and Gynecology, Women and Children’s Hospital of Chongqing Medical University, Chongqing Health Center for Women and Children, Chongqing, China; ^4^ Department of Clinical Research Center, Women and Children’s Hospital of Chongqing Medical University, Chongqing Health Center for Women and Children, Chongqing, China; ^5^ Department of Quality Management, Women and Children’s Hospital of Chongqing Medical University, Chongqing Health Center for Women and Children, Chongqing, China

**Keywords:** BMI, gestational depression, depressive phenotypes, pregnancy, obesity

## Abstract

**Objective:**

To investigate the association between pre-pregnancy body mass index (BMI) and gestational depressive phenotypes.

**Methods:**

The pregnant women receiving the first prenatal examination (4th –13th week of gestation) in Chongqing Health Center for Women and Children were recruited between February 2020 and September 2021. Depressive phenotypes was assessed by the Patient Health Questionnaire (PHQ-9) and the Symptom Checklist 90 (SCL-90) scale at recruitment. Pre-pregnancy weight and height were self-reported by the participants. Demographic and obstetric characteristics were obtained from the hospital information system. The association between pre-pregnancy BMI and the scores of PHQ-9 or SCL-90 scale was investigated by uni-variate analysis with Kruskal-Wallis test and by multi-variate analysis with linear regression model with adjustment of age, parity, smoking, alcohol consumption, and assisted reproduction. The association between pre-pregnancy BMI and PHQ-9 or SCL-90 diagnosed depressive phenotypes was analyzed by Chi-square test and logistic regression respectively.

**Results:**

A total of 12,099 pregnant women were included, where 100% of them filled out the PHQ-9 scale and 99.6% filled out the SCL-90 scale, and 47.26% and 4.62% of the pregnant women had depressive phenotypes, respectively. Women with higher pre-pregnancy BMI had lower depressive phenotypes scores during pregnancy. Multivariable analysis of the PHQ-9 scale showed that overweight/obese subjects had a higher incidence of depressive phenotypes compared with subjects with normal BMI (OR=0.803, 95% CI [0.723, 0.892]). In a stratified analysis assessed by the PHQ-9, women who were overweight/obese prior to pregnancy were less likely to develop depressive phenotypes during pregnancy than women who were normal weight prior to pregnancy, regardless of whether they were nulliparous (OR=0.795, 95%CI[0.696,0.908]) or multiparous (OR=0.809, 95%CI[0.0.681,0.962]), while in the three age groups of 25-29 years, 30-34 years and ≥35 years, pre-pregnancy overweight/obesity were associated with lower risk of gestational depressive phenotypes. However, analysis of the SCL-90 scale showed no statistical association between depressive symptom and BMI. No substantial interaction was observed between BMI and parity or age.

**Conclusions:**

Increased pre-pregnancy BMI may be associated with reduced risk of gestational depressive phenotypes in Chinese women. Independent studies are warranted to validate the findings of the present study.

## 1 Introduction

Pregnant women are susceptive to mental illnesses including depression. The prevalence of depressive mood during pregnancy ranges from 5% to 30% in developed countries (1), and from 18% to 40% in developing countries (2, 3), and the condition in China is similar (4.9% - 39.0%) (4). Studies have confirmed that if the depression during pregnancy cannot be relieved in time, it can not only affect the physiological state, psychology and behavior of pregnant women but the offspring’s infancy, adolescence and adulthood (5). Depression during pregnancy are reported to induce pre-eclampsia, preterm birth, postpartum depression as well as low birth weight and abnormal growth and development in children (5). In addition, the overall cost of perinatal mental illness is significant, the present value of lifetime costs per woman with perinatal depression is £1688 for health and social care, £3028 for productivity and £18,158 for health-related quality of life losses (6). Therefore, it is important to understand the etiology of depression during pregnancy so as to develop targeted interventions to prevent it.

Previous studies have found that depression during pregnancy is related to sociodemographic factors such as age, tobacco smoking, alcohol drinking, parity history et al. (7, 8), and insufficient social support (9). At the same time, some studies have suggested that pre-pregnancy obesity is related to depression during pregnancy. However, the conclusions of these studies so far are inconsistent. A study of pregnant women in Australia (10) found that high pre-pregnancy BMI was associated with an increased risk of prenatal depression and anxiety, and a study in the United States (11) found a strong, positive dose-response association between pre-pregnancy BMI and the likelihood of major depression during pregnancy; Another study in Iran (12) found that the risk of depression in the first prenatal examination for class 2–3 obesity was 3.25-fold greater than normal weight group. However, a study in pregnant women in the United States found no significant correlation between pre-pregnancy BMI and depressive symptoms during pregnancy (13). The authors argued that negative self-directed perceptions about diet, weight and body shape were not very common during pregnancy and may therefore be less likely to affect mood. However, most of these studies focus on European and American populations. Due to the differences in ethnics, economic status and culture, the research conclusions of European and American populations may not be generalizable for Asian population such as Chinese. Therefore, it is necessary to carry out epidemiological studies in the Chinese population to explore whether pre-pregnancy BMI is associated with the occurrence of depressive symptoms during pregnancy.

To investigate whether pre-pregnancy body mass index (BMI) is a predictor of gestational depressive phenotypes of Chinese women, the present study performed an analysis in a representative sample of 12099 pregnant women who attended routine prenatal examination in Chongqing, China.

## 2 Material methods

### 2.1 Participants

**Figure 1:**
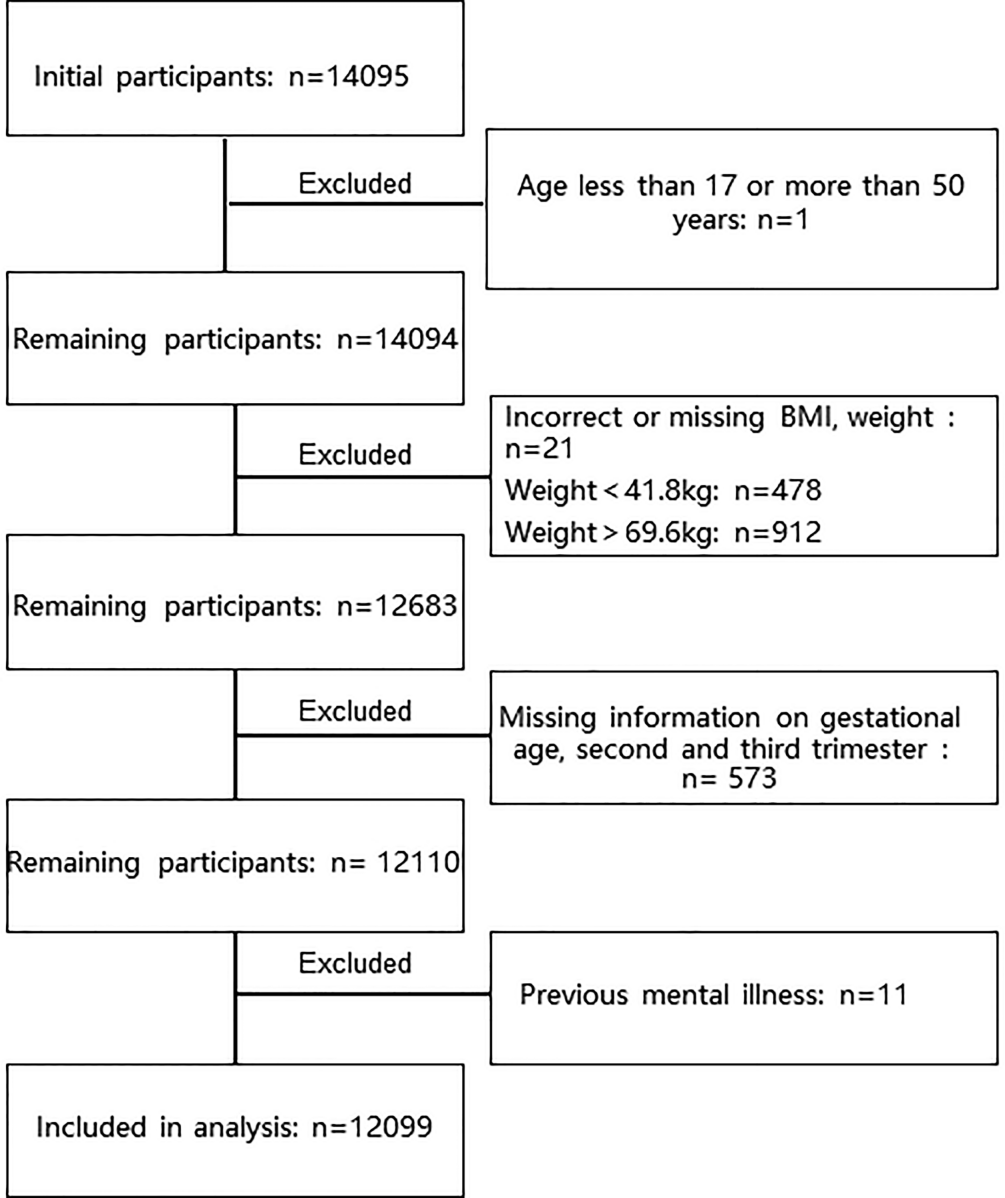
Inclusion and exclusion flowchart.

The present study was conducted from February 2020 to September 2021 at the Chongqing Health Center for Women and Children, China. The Chongqing Health Center for Women and Children is the only the third-class hospital maternal and child health care center in Chongqing. In China, prenatal examination is a national health service with a coverage rate of 96. 2% of mothers in 2014 (14), so using the examination population as the study population is well representative for local mothers. Since February 2020, screening of the psychological status were performed to all the pregnant women who visited for their first prenatal examination of the gestation. As a routine clinical service, psychological nurses used the Patient Health Questionnaire (PHQ-9) and the Symptom Checklist 90 (SCL-90) scale to evaluate the pregnant women’s depressive phenotypes in the obstetric outpatient clinic at their first visit to the hospital during the pregnancy. Participants in the study were just recruited from those women who came for their first prenatal examination during the study period. Those who have the following conditions are excluded:Outside the age range of 15-50 years; Gestational weeks outside of 4-13 weeks; Missing BMI data;Weight outside the range of P5-P95 in Chongqing (15); Past history of mental illness;Missing PHQ-9 or SCL-90 psychological screening results. (**Figure 1**)

### 2.2 Estimation of pre-pregnancy BMI

The height and weight before pregnancy were reported by the participants. Then the pre-pregnancy BMI of each participant was calculated according to the formula: BMI = weight (Kg)/height (m) ^2^. This information was recorded in the hospital information system by the nurses of the obstetric department According to the Chinese standards (16), BMI can be categorized into several groups as following: underweight (BMI < 18.5), normal weight (18.5 ≤ BMI < 24.0), overweight and obesity (BMI ≥ 24.0).

### 2.3 Estimation of depressive phenotypes

The PHQ-9 scale is the first screening tool based on the criteria of the Diagnostic and Statistical Manual of Mental Disorders, Fourth Edition(DSM-IV), which serves the dual purpose of diagnosing depression and assessing the severity of depressive symptoms (17, 18). A score of 0 (not at all) - 3 (almost every day) was assigned to each question. A total score of 0-4 indicates no depression and a score of 5-27 indicates possible depression. (17, p. 9) The SCL-90 scale has a total of 90 items, each of which is scored on a 1-5 scale. The 90 items can be used to calculate 10 factors to reflect a wide range of mental symptoms, including “depression” which was selected by the present study. to evaluate depressive symptoms, Higher score of the factor indicates severer psychological symptoms, and ≥2 was considered as positive (19). The two scales are simple, operational, and validated by domestic and international studies to have high reliability and validity. (20, 21)

### 2.4 Demographic and obstetric characteristics

As a routine clinical process, the following information was collected during the first prenatal examination: age, gestational week, tobacco smoking, alcohol drinking, pregnancy history, parity history, whether with risk factors (http://www.gov.cn/gongbao/content/2018/content_5265003.htm), and whether assisted reproduction technology (ART, Assisted Reproductive Technology) was used. The information was recorded in the maternal information management system, which was designed by the Chinese Center for Disease Control and Prevention and was used around China. In the present study, age was divided into four grades: <25 years old, 25-29 years old, 30-34 years old and ≥35 years old. Pregnancy history was divided into three grades: 1 time, 2 times and ≥3 times. The parity history was divided into two categories: 0 times and ≥1 times.

### 2.5 Statistical analysis

The data was organized and statistically analyzed using SPSS version 26.0 and R version 4.1.1. The association between pre-pregnancy BMI and the scores of PHQ-9 or SCL-90 scale was investigated by uni-variate analysis with Kruskal-Wallis test and by multi-variate analysis with linear regression model with adjustment of age, parity, smoking, alcohol consumption, and assisted reproduction. The association between pre-pregnancy BMI and PHQ-9 or SCL-90 diagnosed depressive phenotypes was analyzed by chi-square test and logistic regression respectively. Furthermore, hierarchial analysis by age, delivery history and pregnancy mode was performed. Interaction analysis was used to compare differences in effect sizes between the different strata. For all participants, only SCL-90 scale scores, tobacco smoking and alcohol drinking have missing data, and since no more than 1.5% of the data were missing in the major analyses, imputation was not used to compensate for missing data. *P*<0.05 was regarded as a statistically significant difference.

### 2.6 Ethics approval

The authors assert that all procedures contributing to this work comply with the ethical standards of the relevant national and institutional committees on human experimentation and with the Helsinki Declaration of 1975, as revised in 2008.

This study had been approved by the Ethics Committee of Chongqing Health Center for Women and Children (No.2018-20-2). Informed consent was obtained from all the participants.

## 3 Results

### 3.1 General demographics

A total of 12,099 study participants were included in the analysis, with 45(0.37%) participants missing data of the SCL-90 scale. The mean age of the participants was 30.04 ± 4.24 years, with 11080 (91.58%) participants were ≥ 25 years old. Most women were non-smokers (97.41%) and non-drinkers (84.52%), 63.43% of the participants were pregnant for the second time or more, 99.79% had high risk factors and 86.06% were pregnant spontaneously. According to the PHQ-9 scale, the mean score of participants was 4.99 ± 3.73, with 47.3% of participants indicating possible depressive phenotypes, while according to the SCL-90 scale, the mean score was 1.29 ± 0.33 and only 4.6% of participants were estimated to have depressive phenotypes.

### 3.2 Uni-variate analysis

Uni-variate analysis for the association between pre-pregnancy BMI and the results of the two depressive scales were done by chi-square test (for scale score) and Kruskal-Wallis test (for positive phenotype). Similar analyses were also done for the potential confounders including age, season, tobacco smoking, alcohol drinking, pregnancy history, parity history, high risk factors during pregnancy, and ART medication.

For the PHQ-9 scale, 50.70% of the participants in the underweight group were in depressive phenotypes, and their mean score was 8.05 ± 3.37; 47.40% of the participants in the normal group were in depressive phenotypes, and their mean score was 7.94 ± 3.30; 41.82% of the participants in the overweight or obese group were in depressive phenotypes, and their mean score was 7.75 ± 3.34, and there were significant group differences in PHQ-9 scores by pre-pregnancy BMI grouping (p<0.001). The Kruskal-Wallis test showed that age, tobacco smoking, alcohol drinking, parity history, whether or not ART pre-pregnancy BMI were all associated with PHQ-9 scores; the chi-square test showed that age, tobacco smoking, alcohol drinking, parity history, whether or not ART and pre-pregnancy BMI were all associated with PHQ-9 scores; (**Table 1**). The results represented a linear rather than U-shaped pattern of relationship between pre-pregnancy BMI and PHQ-9 score and PHQ-9 estimated depressive phenotypes. The results of classifying BMI according to WHO standards see **Table S1** in the Appendix.

**Table 1:** The distribution of depressive phenotypes estimated by the PHQ-9 and the score of the scale.

Characteristics		NO.	PHQ-9-Score	Incidence of depressive phenotypes		*P^a^*	*P^b^*
				Depressive women	Non- depressive women		
BMI	<18.5	2495(20.62%)	5.27 ± 3.81	1265(50.70%)	1230(49.30%)	<0.01	<0.01
	18.5-24.0	7825(64.67%)	5.00 ± 3.73	3709(47.40%)	4116(52.60%)		
	≥24.0	1779(14.70%)	4.59 ± 3.60	744(41.82%)	1035(58.18%)		
Age	<25	1019(8.42%)	5.58 ± 4.06	541(53.09%)	478(46.91%)	<0.01	<0.01
	25-30	4771(39.43%)	5.04 ± 3.63	2305(48.31%)	2466(51.69%)		
	30-35	4451(36.79%)	4.94 ± 3.73	2089(46.93%)	2362(53.07%)		
	≥35	1858(15.36%)	4.67 ± 3.76	783(42.14%)	1075(57.86%)		
Season	Spring	4052(33.49%)	4.92 ± 3.64	1929(47.61%)	2123(52.93%)	0.41	0.68
	Summer	3923(32.42%)	5.10 ± 3.83	1872(47.72%)	2051(52.28%)		
	Autumn	2210(18.27%)	5.01 ± 3.78	1028(46.52%)	1182(53.48%)		
	Winter	1914(15.82%)	4.91 ± 3.65	889(46.45%)	1025(53.55%)		
Tobacco smoking	No	11703(97.41%)	4.96 ± 3.70	5499(46.99%)	6204(53.01%)	<0.01	<0.01
	Yes	311(2.59%)	6.24 ± 4.64	177(56.91%)	134(43.09%)		
Alcohol drinking	No	10073(84.52%)	4.90 ± 3.70	4676(46.42%)	5397(53.58%)	<0.01	<0.01
	Yes	1845(15.48%)	5.43 ± 3.83	941(51.00%)	904((49.00%)		
Pregnancy history	1	4423(36.56%)	4.88 ± 3.59	2033(45.96%)	2390(54.04%)	0.19	0.10
	2	3271(27.03%)	4.99 ± 3.65	1571(48.03%)	1700(51.97%)		
	≥3	4405(36.40%)	5.11 ± 3.93	2114(47.99%)	2291(52.01%)		
Parity history	0	8165(67.48%)	5.09 ± 3.73	3959(48.49%)	4206(51.51%)	<0.01	<0.01
	≥1	3934(32.52%)	4.78 ± 3.73	1759(44.71%)	2175(55.29%)		
High risk factors	No	26(0.21%)	3.65 ± 2.31	11(42.13%)	15(57.69%)	0.14	0.61
	Yes	12073(99.79%)	5.00 ± 3.74	5707(47.27%)	6366(52.73%)		
ART	No	10413(86.06%)	3.65 ± 2.31	4967(47.70%)	5446(52.30%)	<0.01	0.02
	Yes	1686(13.94%)	5.00 ± 3.74	751(44.54%)	935(55.46%)		
Total		12099	4.99 ± 3.73	5718(47.26%)	6381(52.74%)		

^a^The difference of depressive phenotypes incidence was analyzed by Kruskal-Wallis test.

^b^The difference of PHQ-9 score was analyzed by Chi-square test. Colored text represent the seasons when pregnant women filled out the PHQ-9 scale and the SCL-90 scale at their first hospital visit.

However, for the SCL-90 scale, only 4.54% of the participants in the underweight group were in depressive phenotypes, and their mean score was 2.42 ± 0.43; 4.70% of the participants in the normal group were in depression, and their mean score was 2.38 ± 0.43; 4.34% of the participants in the overweight or obese group were in depression, and their mean score was 2.39 ± 0.47, and there were no significant group differences in PHQ-9 scores by pre-pregnancy BMI grouping (*P*=0.79). The Kruskal-Wallis test showed that age, tobacco smoking, alcohol drinking, pregnancy history and parity history were all associated with scores on the SCL-90 scores; the chi-square test showed that age, tobacco smoking, alcohol drinking, parity history, were all associated with scores on the SCL-90 scores (**Table 2**). The results of classifying BMI according to WHO standards see **Table S2** in the Appendix.

**Table 2:** The distribution of depressive phenotypes estimated by the SCL-90 and the score of the scale.

Characteristics		NO.	SCL-90-Score	Incidence of depressive phenotypes		*P^a^*	*P^b^*
				Depressive women	Non- depressive women		
BMI	<18.5	2489(20.66%)	1.29 ± 0.34	113(4.54%)	2376(95.46%)	0.13	0.79
	18.5-24.0	7782(64.61%)	1.29 ± 0.33	366(4.70%)	7416(95.30%)		
	≥24.0	1774(14.73%)	1.28 ± 0.33	77(4.34%)	1697(95.66%)		
Age	<25	1015(8.43%)	1.35 ± 0.41	88(8.67%)	927(91.33%)	<0.01	<0.01
	25-30	4753(39.46%)	1.29 ± 0.32	196(4.12%)	4557(95.88%)		
	30-35	4428(36.76%)	1.28 ± 0.33	191(4.31%)	4237(95.69%)		
	≥35	1849(15.35%)	1.27 ± 0.32	81(4.38%)	1768(95.62%)		
Season	Spring	4052(33.64%)	1.28 ± 0.32	170(4.20%)	3882(95.80%)	0.30	0.11
	Summer	3923(32.57%)	1.30 ± 0.35	207(5.28%)	3716(94.72%)		
	Autumn	2210(18.35%)	1.29 ± 0.34	100(4.52%)	2110(95.48%)		
	Winter	1860(15.44%)	1.28 ± 0.31	79(4.25%)	1781(95.75%)		
Tobacco smoking	No	11653(97.43%)	1.28 ± 0.33	517(4.44%)	11136(95.56%)	<0.01	<0.01
	Yes	307(2.57%)	1.43 ± 0.48	34(11.07%)	273(88.93%)		
Alcohol drinking	No	10034(84.57%)	1.28 ± 0.33	429(4.28%)	9605(95.72%)	<0.01	<0.01
	Yes	1831(15.43%)	1.32 ± 0.36	115(6.28%)	1716(93.72%)		
Pregnancy history	1	4397(36.50%)	1.28 ± 0.32	192(4.37%)	4205(95.63%)	0.02	0.10
	2	3260(27.07%)	1.29 ± 0.34	138(4.23%)	3122(95.77%)		
	≥3	4388(36.43%)	1.30 ± 0.35	226(5.15%)	4162(94.85%)		
Parity history	0	8124(67.45%)	1.30 ± 0.34	410(5.05%)	7714(94.95%)	<0.01	<0.01
	≥1	3921(32.55%)	1.26 ± 0.31	146(3.72%)	3775(96.82%)		
High risk factors	No	25(0.21%)	1.17 ± 0.12	0	25(100%)	0.24	0.63^c^
	Yes	12020(99.79%)	1.29 ± 0.33	556(4.63%)	11464(95.37%)		
ART	No	10372(86.11%)	1.30 ± 0.34	486(4.69%)	9886(95.31%)	0.62	0.36
	Yes	1673(13.89)	1.28 ± 0.31	70(4.18%)	1603(95.82%)		
Total		12045	1.29 ± 0.33	556(4.62%)	11489(95.38%)		

^a^The difference of depressive phenotypes incidence was analyzed by Kruskal-Wallis test.

^b^The difference of PHQ-9 score was analyzed by Chi-square test.

^c^The difference of depressive phenotypes incidence was analyzed by Fisher’s precision probability test. Colored text represent the seasons when pregnant women filled out the PHQ-9 scale and the SCL-90 scale at their first hospital visit.

### 3.3 Multivariable analysis

For the PHQ-9 scale, after adjustment of age, tobacco smoking, alcohol drinking, parity history and ART medication by linear regression, each unit increase in pre-pregnancy BMI was associated with 0.065 (95% CI:-0.091, -0.039, *P*<0.01) lower score of the PHQ-9 scale. Logistic regression also showed that pre-pregnancy BMI grade was significantly associated with the risk of depressive phenotypes (OR=0.976, 95% CI: 0.953, 0.981, *P*<0.01). Compared to the participants with normal weight, the risk of depressive phenotypes was 0.803 fold (95% CI: 0.723, 0.892, *P*<0.01) for the overweight/obese group, while the risk for the underweight group was 1.076 fold but not statistically significant (95% CI: 0.979, 1.182, *P*=0.13; see **Table 3**).

**Table 3:** The association between pre-pregnancy BMI and depressive phenotypes: Multivariable analysis.

		PHQ-9				SCL-90			
		OR/β	lower limit	upper limit	*P*	OR/β	lower limit	upper limit	*P*
linear regression	BMI	-0.065	-0.091	-0.039	<0.01	-0.001	-0.003	0.002	0.62
	Underweight V.S. normal weight	-0.044	-0.083	-0.004	0.03	0.002	-0.002	0.005	0.40
	Overweight/obese V.S. normal weight	-0.072	-0.105	-0.039	<0.01	-0.002	-0.005	0.001	0.17
	BMI ^a^	0.967	0.953	0.981	<0.01	1.002	0.969	1.036	0.92
logistic regression	Underweight V.S. normal weight	1.076	0.979	1.182	0.13	0.848	0.675	1.065	0.16
	Overweight/obese V.S. normal weight	0.803	0.723	0.892	<0.01	0.922	0.714	1.191	0.54

^a^Inclusion of BMI as a continuous variable in logistic regression

As for the SCL-90 scale, after adjustment of the potential confounders, the logistic regression analysis showed that pre-pregnancy BMI was not associated with the risk of depressive phenotypes (OR=1.002, 95% CI: 0.969, 1.036, *P*=0.92). No significant association between pre-pregnancy BMI and SCL-90 score was observed by the linear regression analysis, either (β=-0.001, 95% CI: -0.003, 0.002, *P*=0.62; see **Table 3**)

### 3.4 Hierarchical analysis and interaction

**Figure 2:**
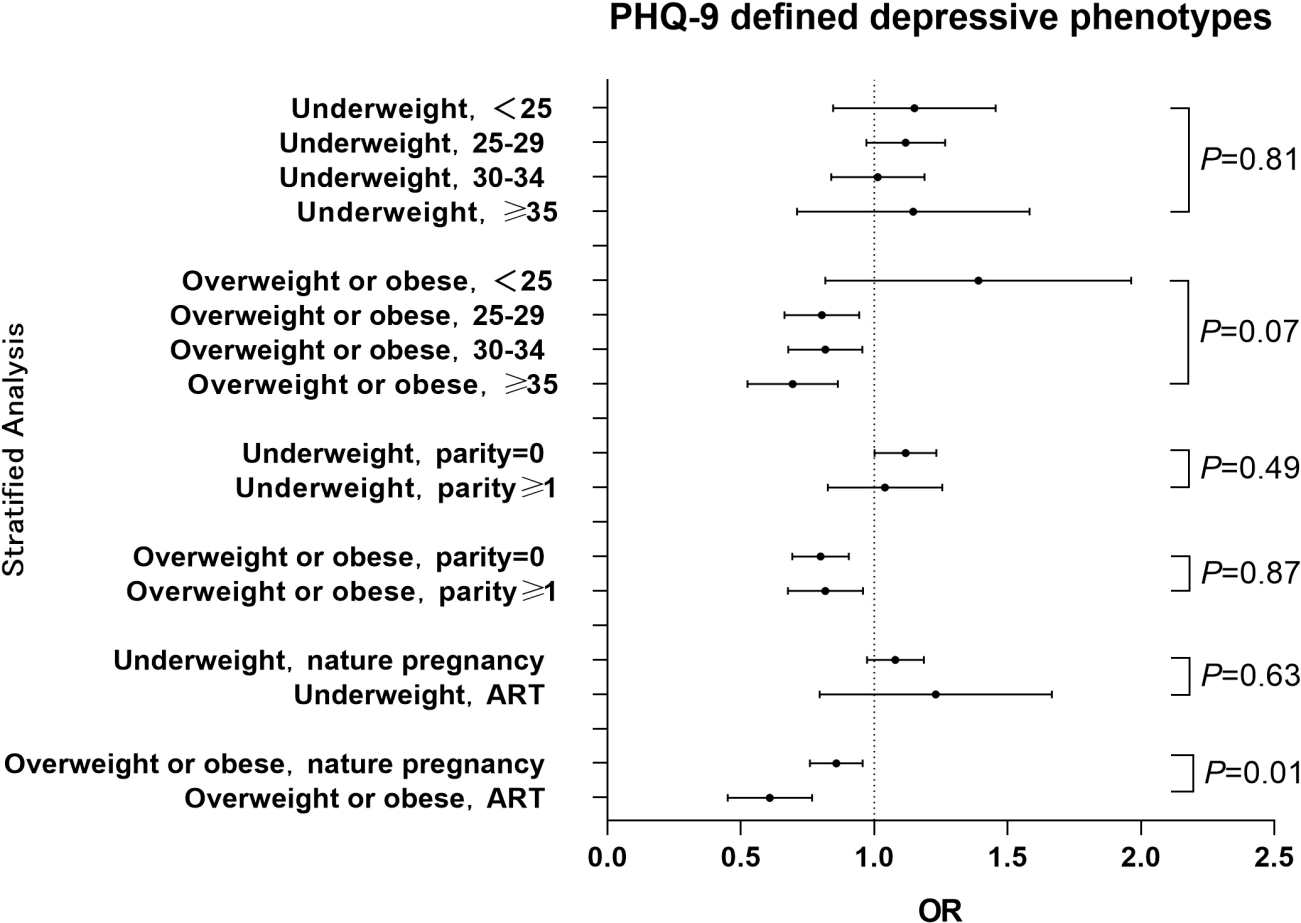
Hierarchical analysis of the association between pre-pregnancy BMI and PHQ-9 estimated depressive phenotypes among different stratums of age, parity history or way of conception The OR value for each stratum was calculated with comparison to the women with normal BMI. Pint refers to P for interaction.

The relationships between pre-pregnancy BMI and PHQ-9 score and PHQ-9 estimated depressive phenotypes were investigated in a hierarchical analysis by age, parity and way of conception respectively. The results of the hierarchical analysis by age showed that pre-pregnancy BMI was associated with gestational depressive phenotypes in the 25-29 years (*P*<0.01), 30-34 years (*P*=0.05) and ≥35 years (*P*<0.01) groups, while no significant association was observed in the <25 years group (*P*=0.40). The results of the parity hierarchical analysis showed that pre-pregnancy BMI was associated with gestational depressive phenotypes in both primiparous (*P*<0.01) and transmaternal (*P*=0.05) women. Hierarchical analysis by way of conception showed that pre-pregnancy BMI was associated with gestational depressive phenotypes in both natural pregnancy women (*P*<0.01) and ART medication (*P*<0.01) women. The interaction analysis between age and pre-pregnancy BMI showed a significant difference in OR by age in the overweight or obese group (*P*=0.07) and no significant difference in the underweight group (*P*=0.81). Interaction analysis of parity with pre-pregnancy BMI showed no significant difference in OR between primiparous and transmaternal women in both the overweight or obese group (*P*=0.87) and the underweight group (*P*=0.49). The interaction analysis between way of conception and pre-pregnancy BMI showed a significant effect in the overweight or obese group (*P*=0.01) but not in the underweight group (*P*=0.63) (**Figure 2**) The results of classifying BMI according to WHO standards see **Figure S1** in the Appendix.

**Figure 3:**
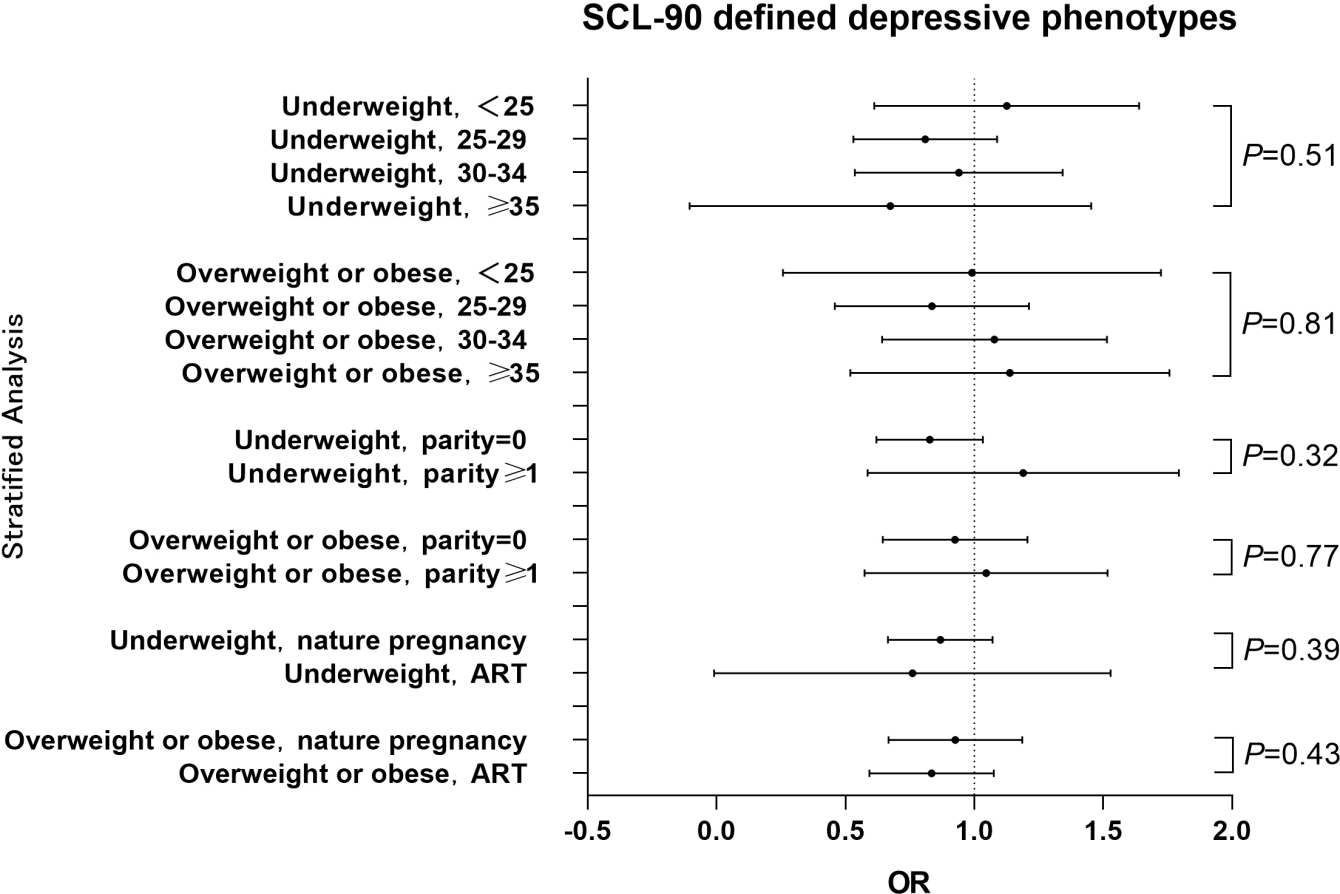
Hierarchical analysis of the association between pre-pregnancy BMI and SCL-90 estimated depressive phenotypes among different stratums of age, parity history or way of conception The OR value for each stratum was calculated with comparison to the women with normal BMI. Pint refers to P for interaction.

A hierarchical analysis of the SCL-90 scale by age, parity history and way of conception showed that pre-pregnancy BMI was not statistically associated with gestational depressive phenotypes across all age, parity history and way of conception groups. Interaction analysis showed no interaction between age and pre-pregnancy BMI, and no interaction between history of births and pre-pregnancy BMI. (**Figure 3**) The results of classifying BMI according to WHO standards see **Figure S2** in the Appendix.

## 4 Discussion

Based on the analysis of 12099 Chinese pregnant women, the results of the present study showed that pre-pregnancy BMI was associated with gestational depressive phenotypes which was estimated by the PHQ-9 scale. Compared to the women with normal weight, the underweight women was associated with 1.076 (95%CI:0.970 to 1.182) fold risk of depression, while the overweight or obese women was associated with 0.803 (95%CI:0.720 to 0.892) fold risk. However, when estimated by the SCL-90 scale, the prevalence of depressive phenotypes was substantially lower in the population (4.62% by SCL-90 versus 47.26% by PHQ-9), and no significant association was found between pre-pregnancy BMI and depressive phenotypes. Hierarchical analysis based on the PHQ-9 scale showed that the pre-pregnancy overweight and obesity were inversely associated with depressive phenotypes in most age groups, and there was a suggestive interaction between age and pre-pregnancy BMI, where overweight/obese at higher age tend to have a lower risk of depressive phenotypes. Either for natural pregnancy women or for ART patients, pre-pregnancy overweight or obese was associated with a lower risk of depressive phenotypes. No substantial interaction between parity history and pre-pregnancy BMI was found.

The present study found that the pre-pregnancy overweight or obesity group had a lower risk of depressive phenotypes during pregnancy compared with the normal weight group, which was inconsistent with the findings of Carol Shieh (10) et al. and Charlotte V. Farewell (22) et al. who concluded that pre-pregnancy BMI was positively associated with gestational depressive phenotypes. In contrast, the studies by Alice S. Carter (13) et al. and Nafisa Insan (23) et al. argued that pre-pregnancy BMI was not associated with gestational depressive phenotypes. We speculate that the possible reason is as follows: the populations of the previous studies were all from western countries, whereas the participants in our study were Chinese. The traditional Chinese culture encourages pregnant women to eat more and gain higher weight to ensure adequate nutrition for the fetal growth, without a negative attitude to overweight or obesity, while lean body shape is not favorable. This difference of traditional culture may have led to a lesser psychological stress about increased body shape to pregnancy women in China compared to those in western countries. In addition, our study also found that pre-pregnancy overweight/obesity was inversely associated with depressive phenotypes during pregnancy in the 25-29, 30-34, and ≥35 age groups, but not in the <25-year-old age group. This result of interaction analysis also supported the above hypothesis that the effect size of overweight/obesity seemed larger in the women with higher age compared to the younger women (**Figure 2**), as younger women are less affected by traditional ideas and are more likely to accept modern medical knowledge of body shape control. Furthermore, hierarchical analysis revealed that pre-pregnancy overweight or obesity was associated with depression phenotype in the natural pregnancy and ART medication groups, not in the underweight group. An interaction between way of conception and pre-pregnancy BMI was observed, which is consistent with the results of previous studies (24, 25). This may be a result of a more positive attitude towards pregnancy in those who received ART medication and became pregnant, thus becoming more comfortable with the state of pregnancy (26).

The different patterns of BMI- depressive phenotypes relationship between the present study and the studies of western populations may suggest that social circumstance and self-evaluation about body shape are the mechanism by which pre-pregnancy BMI influences the occurrence of gestational depressive phenotypes (27). A study in north-east England noted that the pregnant women’s self-assessment of their weight gain led to distinct feelings among different individuals. Some pregnant women reported that some weight gain was acceptable in order to have a healthy, normal-weight baby, while others reported negative feelings about significant weight gain (28). In a study in Taiwan, the subject reported that all the changes that occurred in her body were a result of her new role as a mother, and that the weight gain may provide assurance of fetal health. The researchers interpreted that pregnancy provided a temporary opportunity for pregnant women to stop associating thinness strictly with femininity. Being pregnant also brings with them a unique standard of happy, fulfilled, self-sacrificing mother-to-be. (29). This is also in concordance with the traditional culture widely accepted in China. To be noted, the prevalence of obesity is much lower in Chinese pregnant women than it is in western countries (30). This may have brought different psychological effects to the two populations. However, as the sample size of obese women (n=62) in our study is too small, separate analysis for the relationship of obesity and depressive phenotypes is not feasible. Our result may mainly reflect the effect of mild increase in body weight. Another possible biological mechanism is that the higher consumption of carbohydrates in overweight or obese individuals may have reduced the occurrence of depressive symptoms. It is well known that carbohydrates temporarily alleviate the vegetative symptoms of depression by increasing central serotonergic activity, while contributing to weight gain (31). As pregnant women in China bear relatively smaller stress of weight gain, the net benefit effect on emotion caused by carbohydrates could be more noticeable.

The difference between women with depressive phenotypes screened by the SCL-90 scale and the PHQ-9 scale was very large in our sample. The reason may be that the PHQ-9 scale can be used not only as a criteria-based diagnostic indicator of depression, but also as a reliable and valid indicator of the severity of depression (32). The SCL-90 scale is used essentially to measure the severity of self-perceived symptoms in outpatients, rather than as a mass screening in the general population (33), and the depression section of it reflects a wide spectrum of concepts linked to clinical clusters of depressive symptoms (34). The finding that pre-pregnancy BMI was associated with PHQ-9 but not SCL-90 estimated depressive phenotype may suggest that body shape affects Chinese women’s depressive symptom rather than the development of a serve disease.

There are still some shortcomings in this study. First, this is an observational study, and causal inference cannot be made. Second, all participants of the present study were recruited from one hospital. It may not well represent the childbearing age Chinese women. Third, the study used self-reported height and weight, which may have recall bias, although first trimester is not far from pre-pregnancy period. Fourth, the PHQ-9 and SCL-90 scales were not questionnaires designed specifically for pregnant women. The relationship of pre-pregnancy BMI and clinical-diagnosed depressive phenotypes in Chinese women deserves further investigation. Finally, the present study only recruited women in the first trimester, making it difficult to extrapolate the results to the women of second and third trimesters.

## 5 Conclusion

Based on a large sample size of Chinese women, the present study suggests that pre-pregnancy BMI is associated with the risk of depressive phenotypes during pregnancy. Among pregnant women aged ≥25 years, an increase in pre-pregnancy BMI was associated with a reduced risk of depressive phenotypes during pregnancy, and there was a suggestive interaction between age and BMI, where the effect size of overweight/obese seemed larger in the women with higher age. Further research is necessary to validate the relationship between pre-pregnancy BMI and depressive phenotypes during pregnancy for Chinese women.

## Data availability statement

The original contributions presented in the study are included in the article/**Supplementary Material**. Further inquiries can be directed to the corresponding authors.

## Ethics statement

The studies involving human participants were reviewed and approved by The authors assert that all procedures contributing to this work comply with the ethical standards of the relevant national and institutional committees on human experimentation and with the Helsinki Declaration of 1975, as revised in 2008. This study had been approved by the Ethics Committee of Chongqing Health Center for Women and Children (No.201820-2). Informed consent was obtained from all the participants. Written informed consent to participate in this study was provided by the participants’ legal guardian/next of kin.

## Author contributions

QC, HZ and JC designed research. YC, HG, NZ and WZ conducted research. YC analyzed the data. YC, HG and QC wrote the paper. QC and JC, HZ had primary responsibility for final content. All authors contributed to the article and approved the submitted version.
