# Supplementary material for: Pre-pregnancy BMI was associated with gestational depressive phenotypes in a population of 12,099 women in Chongqing, China
Source: Front Endocrinol (Lausanne). 2023 Jan 10;13:1058160. doi: 10.3389/fendo.2022.1058160 (PMC9871462; doi:10.3389/fendo.2022.1058160)
Supplement: Supplementary file 1 [file DataSheet_1.doc]

**Supplementary material**

Table S1 The distribution of depressive phenotypes estimated by PHQ-9 and scores on the scale (BMI classification from WHO)

| Characteristics | | Number of participants | Score | | Incidence of depression | | | *P^a^* | | | *P^b^* |
| --- | --- | --- | --- | --- | --- | --- | --- | --- | --- | --- | --- |
|  |  |  |  |  | Depressive women | Non- depressive women | |  |  |  |  |
| BMI | ＜18.5 | 2494（20.62%） | 5.27±3.81 | 1264（50.70%） | | | 1230(49.30%) | | ＜0.01 | ＜0.01 | |
|  | 18.5-25.0 | 8523（70.44%） | 4.97±3.74 | 4011（52.9%） | | | 4512(52.90%) | |  |  |  |
|  | ≥25.0 | 1082（8.94%） | 4.53±3.46 | 639（59.1%） | | | 443(40.9%) | |  |  |  |

^a^: analyzed by Chi-square test

^b^: analyzed by Kruskal-Wallis test

Table S2 The distribution of depressive phenotypes estimated by SCL-90 and scores on the scale (BMI classification from WHO)

| Characteristics | | Number of participants | Score | | Incidence of depressive symptoms | | | | *P^a^* | *P^b^* | | |
| --- | --- | --- | --- | --- | --- | --- | --- | --- | --- | --- | --- | --- |
|  |  |  |  |  | Depressive women | | Non- depressive women | |  |  |  |  |
| BMI | ＜18.5 | 2488(20.66%) | 1.29±0.34 | 101(4.1%) | | 2387(95.9%) | | 0.18 | | | 0.16 |  |
|  | 18.5-25.0 | 8477(70.38%) | 1.29±0.34 | 334(3.9%) | | 8134(96.1%) | |  |  |  |  |  |
|  | ≥25.0 | 1080(8.97%) | 1.27±0.31 | 31(2.9%) | | 1049(97.1%) | |  |  |  |  |  |

^a^: analyzed by Chi-square test

^b^: analyzed by Kruskal-Wallis test

|  | Table S3 Multivariate Analysis (BMI classification from WHO) | | | | | | | | | | |  |
| --- | --- | --- | --- | --- | --- | --- | --- | --- | --- | --- | --- | --- |
|  | |  | PHQ-9 | | | |  | SCL-90 | | | | |
|  | |  | OR/β | lower limit | upper limit | *P* |  | OR/β | lower limit | upper limit | *P* | |
| linear regression | | BMI | -0.065 | -0.091 | -0.039 | ＜0.01 |  | -0.001 | -0.003 | 0.002 | 0.62 | |
|  |  | Underweight vs normal weight | -0.050 | -0.084 | -0.016 | ＜0.01 |  | 0.002 | -0.001 | 0.005 | 0.25 | |
|  |  | Overweight/ obese vs normal weight | -0.072 | -0.105 | -0.039 | ＜0.01 |  | -0.002 | -0.005 | 0.001 | 0.17 | |
| logistic regression | | BMI ^a^ | 0.967 | 0.953 | 0.981 | ＜0.01 |  | 1.002 | 0.969 | 1.036 | 0.92 | |
|  |  | Underweight vs normal weight | 1.086 | 0.989 | 1.193 | 0.08 |  | 0.911 | 0.717 | 1.159 | 0.45 | |
|  |  | Overweight/ obese vs normal weight | 0.774 | 0.68 | 0.882 | ＜0.01 |  | 0.922 | 0.484 | 1.037 | 0.08 | |

^a^: Included only underweight and normal weight women


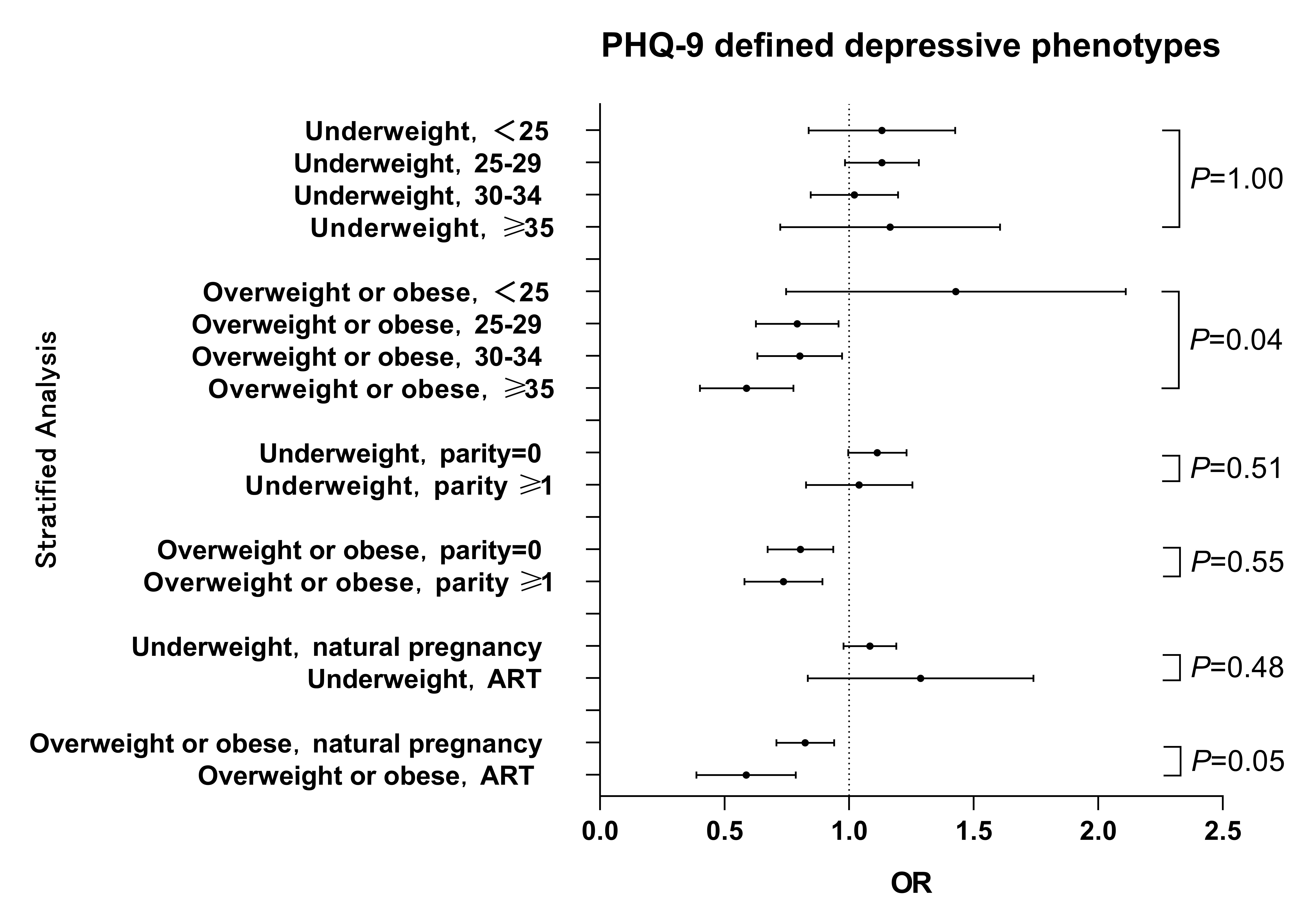


Figure S1 Hierarchical analysis of the association between pre-pregnancy BMI and PHQ-9 estimated depressive phenotypes among different stratums of age, parity history or way of conception (BMI classification from WHO)

The OR value for each stratum was calculated with comparison to the women with normal BMI. Pint refers to P for interaction.


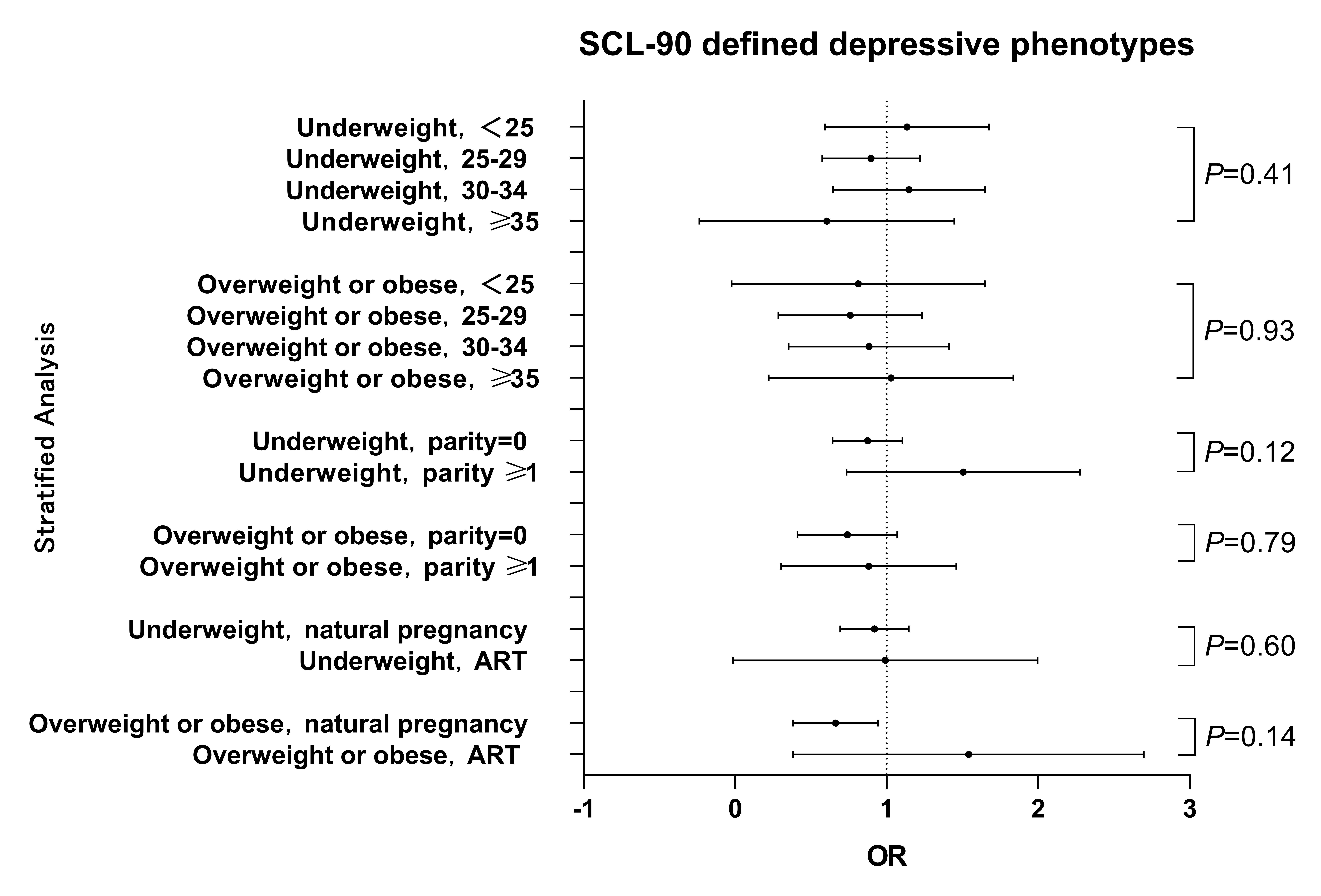


Figure S2 Hierarchical analysis of the association between pre-pregnancy BMI and SCL-90 estimated depressive phenotypes among different stratums of age, parity history or way of conception (BMI classification from WHO)

The OR value for each stratum was calculated with comparison to the women with normal BMI. Pint refers to P for interaction.
